# TEPITOPEpan: Extending TEPITOPE for Peptide Binding Prediction Covering over 700 HLA-DR Molecules

**DOI:** 10.1371/journal.pone.0030483

**Published:** 2012-02-23

**Authors:** Lianming Zhang, Yiqing Chen, Hau-San Wong, Shuigeng Zhou, Hiroshi Mamitsuka, Shanfeng Zhu

**Affiliations:** 1 School of Computer Science and Shanghai Key Lab of Intelligent Information Processing, Fudan University, Shanghai, China; 2 Department of Computer Science, City University of Hong Kong, Hong Kong, China; 3 Bioinformatics Center, Institute for Chemical Research, Kyoto University, Gokasho, Uji, Japan; Kyushu Institute of Technology, Japan

## Abstract

**Motivation:**

Accurate identification of peptides binding to specific Major Histocompatibility Complex Class II (MHC-II) molecules is of great importance for elucidating the underlying mechanism of immune recognition, as well as for developing effective epitope-based vaccines and promising immunotherapies for many severe diseases. Due to extreme polymorphism of MHC-II alleles and the high cost of biochemical experiments, the development of computational methods for accurate prediction of binding peptides of MHC-II molecules, particularly for the ones with few or no experimental data, has become a topic of increasing interest. TEPITOPE is a well-used computational approach because of its good interpretability and relatively high performance. However, TEPITOPE can be applied to only 51 out of over 700 known HLA DR molecules.

**Method:**

We have developed a new method, called TEPITOPEpan, by extrapolating from the binding specificities of HLA DR molecules characterized by TEPITOPE to those uncharacterized. First, each HLA-DR binding pocket is represented by amino acid residues that have close contact with the corresponding peptide binding core residues. Then the pocket similarity between two HLA-DR molecules is calculated as the sequence similarity of the residues. Finally, for an uncharacterized HLA-DR molecule, the binding specificity of each pocket is computed as a weighted average in pocket binding specificities over HLA-DR molecules characterized by TEPITOPE.

**Result:**

The performance of TEPITOPEpan has been extensively evaluated using various data sets from different viewpoints: predicting MHC binding peptides, identifying HLA ligands and T-cell epitopes and recognizing binding cores. Among the four state-of-the-art competing pan-specific methods, for predicting binding specificities of unknown HLA-DR molecules, TEPITOPEpan was roughly the second best method next to NETMHCIIpan-2.0. Additionally, TEPITOPEpan achieved the best performance in recognizing binding cores. We further analyzed the motifs detected by TEPITOPEpan, examining the corresponding literature of immunology. Its online server and PSSMs therein are available at http://www.biokdd.fudan.edu.cn/Service/TEPITOPEpan/.

## Introduction

Major histocompatibility complex (MHC) molecules play a crucial role in the adaptive immune system mediated by T cells [1], in which peptide fragments derived from pathogens first bind to MHC molecules and are then presented on the surface of a cell for recognition by T cell receptor (TCR). This process enables the immune system to detect the presence of foreign pathogens, and thus induce the immune response to eliminate invading pathogens. Accurate identification of peptides that bind to specific MHC molecules is therefore of great importance for the following points: 1) understanding the underlying mechanism of immune recognition; 2) developing effective peptide-based vaccines against infectious diseases; and 3) promising immunotherapies for allergy, autoimmunity, and cancers [2]. In contrast to biochemical experiments that takes lots of expenses and time, computational approaches for predicting MHC binding peptides have received extensive attentions [3], [4]. They have been utilized to select a small number of promising candidate epitopes for further experimental verification [5].

According to their different roles in the immune system, MHC molecules can be divided into two major classes: MHC class I (MHC-I) and MHC class II (MHC-II). MHC-I molecules sample and bind intracellular antigenic peptides (normally nine amino acids), and present them to cytotoxic T lymphocytes (CTL) to stimulate cellular immunity, while MHC-II molecules sample and bind extracellular antigenic peptides (typically 11-20 amino acids), which are presented to helper T lymphocytes [1]. An MHC molecule has the binding groove of nine pockets (or polymorphic cavities), while a binding peptide has a binding core, usually a nonamer, fitted to the binding groove, where one residue of the binding core corresponds to one pocket. The binding groove of MHC-I is closed at both ends, whereas that of MHC-II is open at both ends, which leads to the flexibility in the length and binding core location of MHC-II binding peptide. This difference makes the problem of predicting peptides binding to MHC-II more challenging than that to MHC-I [6]–[9]. In fact, the current state-of-the-art performance of this problem for MHC-I reaches an AUC (Area Under the ROC Curve) of between 0.85 and 0.95, whereas the AUC of the same problem for MHC-II stays roughly between 0.70 and 0.85 [10]. In this work, we focus on predicting MHC-II binding peptides, a more challenging problem.

MHC molecules are highly polymorphic, and there are thousands of MHC allelic variants. For human beings, MHC is known as Human Leukocyte Antigens (HLA), which involves three types of HLA class II molecules: DP, DQ and DR [1]. By June 2011, up to 1457 HLA class II alleles are collected in IMGT/HLA [11]. Each HLA-II molecule consists of two types of domains, 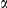 (A) and 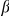 (B). For example, the most widely studied HLA-DR molecules have DRA and DRB, corresponding to 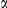 and 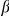 domains, respectively. While DRB is diverse, DRA is almost identical, by which the binding specificities of DR molecules are mainly determined by DRB. Thus, hereafter, the binding specificity of a DRB allele indicates that of the corresponding HLA-DR.

Each MHC molecule has its own binding specificity, meaning that a set of peptides binding to an MHC molecule can be different from those to another MHC molecule. Thus, conventional prediction models are allele-specific, where for a target MHC-II molecule, a model is trained by peptide sequences binding to this molecule to predict the specificity of an arbitrary given peptide [12]–[17]. In general they need 100–200 quantitative peptide-binding measurements for each target molecule [14]. However, the number of MHC-II molecules which can have a large number of binding peptides is very small. For example, IEDB (Jun. 2011), the largest database of MHC binding peptides [18], contains only around 30 HLA-II molecules for which a few hundred peptides have experimentally measured binding affinity. This means that overwhelmingly most MHC-II molecules have very few or even no binding data, which cannot be handled by allele-specific methods. To address this problem, so-called *pan-specific* methods which can predict the specificity of peptides binding to MHC molecules with almost no binders have been developed first for MHC-I [19] and then for MHC-II [20]. These pan-specific methods take into account both peptide sequences and MHC-peptide contact, which is represented by MHC residues being in contact with each binding peptide. The pioneering pan-specific method for HLA-I is MULTIPRED [21], which shares binding data within HLA-I supertype (a group of MHC molecules sharing similar binding specificities [22]) and trained supertype-specific models to cover many HLA-I molecules.

The first pan-specific method for MHC-II molecules is TEPITOPE, which uses position specific scoring matrix (PSSM) [23]. TEPITOPE generates 51 PSSMs, which cover 51 different HLA-DR alleles. These PSSMs are derived from 11 HLA DRB alleles: DRB1*01:01, DRB1*03:01, DRB1*04:01, DRB1*04:02, DRB1*04:04, DRB*07:01, DRB1*08:01, DRB1*11:01, DRB1*13:02, DRB1*15:01 and DRB5*01:01 (the alleles in this paper are represented in current HLA allele nomenclature [24]). There are other five pan-specific methods: MHCIIMulti [25], NetMHCIIpan-1.0 [26], NetMHCIIpan-2.0 [27], MultiRTA [28] and SIADT (Shift Invariant Adaptive Double Threading) [29]. MHCIIMulti is a kernel based method, in which the binding specificity of a target MHC with no binding data can be predicted by using binding data of related MHC alleles. Both NetMHCIIpan-1.0 and NetMHCIIpan-2.0 use ANN (Artificial Neural Network) with co-encoding of both binding peptides and pocket sequences of MHC-II molecules as input. The difference between NetMHCIIpan-1.0 and NetMHCIIpan-2.0 lies in the identification of the peptide binding core. In NetMHCIIpan-1.0, the core is pre-identified using SMM-align [14], whereas in NetMHCIIpan-2.0, the identification of binding core and training weights of ANN are both done in the ANN learning process. MultiRTA considers all possible peptide binding core configurations, and the MHC-peptide binding affinity is computed as a weighted average over the binding affinities of all possible configurations. SIADT is based on threading, which has been used for predicting protein 3D structure. Note that all these methods except SIADT have publicly available implementations. Although TEPITOPE is not necessarily the best in performance among them, TEPITOPE can provide prediction rules which could be easily understood, and thus TEPITOPE has earned wide popularity among biologists [10]. However, a drawback of TEPITOPE is that only 51 DR molecules are covered out of over 700 known DR molecules, which greatly limits its usability.

To overcome this problem, keeping the advantage of TEPITOPE in rule comprehensibility, we propose a new method, TEPITOPEpan, which can extrapolate from the HLA-DR molecules with known binding specificities (PSSMs) in TEPITOPE to the HLA-DR molecules with unknown binding specificities based on pocket similarity. The procedure of TEPITOPEpan for a target HLA is as follows: using the MHC-II HLA-peptide complex structure in Protein Data Bank (PDB), pockets are first represented by the polymorphic residues that have close contact with one or more residues of binding core. Then the pocket similarity between two HLA molecules is computed by the sequence similarity of the corresponding HLA residues. For an uncharacterized HLA-DR molecule, the binding specificity of each pocket was computed as a weighted average of pocket binding specificities over HLA-DR molecules characterized by TEPITOPE. The idea of TEPITOPEpan comes from PickPocket [30], a pan-specific method for MHC-I, which also derived the binding specificities (PSSM) of a novel MHC molecule from a library of specificity matrices (PSSMs). A clear difference between TEPITOPEpan and PickPocket is that TEPITOPEpan uses the library of specificity matrices obtained in TEPITOPE, while PickPocket generates that from binding data directly.

We evaluated the performance of TEPITOPEpan extensively using a variety of datasets, assuming different types of settings, and comparing with the state-of-the-art pan-specific methods. We are especially interested in the performance on uncharaterized MHC molecules, which is the main target of pan-specific methods. Experimental results showed that, for predicting the binding specificities of novel HLA DR molecules, TEPITOPEpan achieved roughly the second best performance next to NetMHCIIpan-2.0, which however cannot show any comprehensible rules. In particular, TEPITOPEpan outperformed competing methods on predicting the location of the binding core. We further checked the obtained rules of TEPITOPEpan by using sequence logos, which showed that primary anchors are well consistent with the literature.

## Materials and Methods

### Data

We generated eight datasets: Nielsen-Set1, Nielsen-Set2, Lin-Set3, Epan-Set4, Bordner-Set5, SYF-Set6, EIEDB-Set7 and EpanCore-Set8. They include various types of peptides, such as MHC binding peptides, endogenously presented MHC ligands, T-cell epitopes, and those obtained from HLA-peptide complexes. We used Nielsen-Set1 to select suitable parameters in TEPITOPEpan, while the remaining seven were used for extensive evaluation, such as comparison with the state-of-the-art pan-specific methods, including NetMHCIIpan-2.0, NetMHCIIpan-1.0 and MultiRTA.

**Nielsen-Set1** which was obtained from [14] has 4,603 peptides with quantitative binding measures covering 14 HLA-DR alleles.

**Nielsen-Set2** was from [27] and comprises 33,931 binding peptides of 24 HLA-DRB alleles.

**Lin-Set3** has 103 overlapping peptides for each of 7 common HLA-DR alleles that were derived from four distinct antigens [9]. Lin-Set3 was downloaded from Dana-Farber Repository for Machine Learning in Immunology (DFRMLI) [31].

**Epan-Set4** has 2,412 peptides associated with 14 novel HLA-DRB alleles (including 2 alleles in Nielsen-Set2 though). Epan-Set4 was generated by us for assessing the predictive performance of competing methods for new alleles, as follows: We first focused on HLA-DRB alleles which were not in the 14 alleles of Nielsen-Set1 and the 11 alleles for TEPITOPE. We then retrieved peptides binding to these HLA-DRB alleles from IEDB (Mar. 2011) [18]. We, for the two alleles (DRB1*03:02 and DRB1*12:01) in Nielsen-Set2, discarded peptides sharing at least nine consecutive residues with a peptide binding to either of the two alleles in Nielsen-Set2. Finally, we kept HLA-DRB alleles with more than 40 peptides, to remove datasets with a small size. Epan-Set4 can be downloaded from TEPITOPEpan website.

**Bordner-Set5** was taken from [28] and has 127 peptides restricted to HLA-DRB1*13:01. Note that Bordner-Set5 is a subset of peptides binding to HLA-DRB1*13:01 in Epan-Set4.

**SYF-Set6** has 1,164 ligands restricted to 28 HLA-DR alleles, from SYFPEITHI (Nov. 2009) [32].

**EIEDB-Set7** comprises 1,325 T-cell epitopes restricted to 42 HLA-DR alleles, being retrieved from IEDB (Jun. 2010) [18]. SYF-Set6 as well as EIEDB-Set7 were prepared to evaluate the competing methods by using HLA-II ligands and T-cell epitopes. SYF-Set6 and EIEDB-Set7 are two datasets in [27]. In these two datasets, all ligands and epitopes in Nielsen-Set2 were eliminated.

**EpanCore-Set8** has 20 distinct 3D complex structures of peptide binding to 7 HLA-DR molecules that have known binding cores and are obtained from PDB, 15 of which are taken from [16]. EpanCore-Set8 was used to evaluate TEPITOPEpan in terms of identifying the peptide binding core.

### Method

TEPITOPE has a library of 11 PSSMs. One PSSM is a 20×9 matrix where nine binding specificity vectors correspond to nine pockets. Each of the 11 PSSMs corresponds to one of 11 known DRB alleles. TEPITOPEpan uses this library to generate a PSSM for an arbitrary HLA-DRB allele. In a generated PSSM, each vector is a weighted average of binding specificity vectors of the corresponding pocket over 11 DRB alleles. The weight can be computed by pocket sequence similarity. Thus the assumption behind TEPITOPEpan is that different alleles have similar binding preferences for one pocket (e.g. P1) if their MHC amino acids for the pocket are similar. The procedure of TEPITOPEpan has the following three steps:

#### Step 1: Generating pseudosequences of MHC binding pockets

We first represent MHC binding pockets by using the 3D structure of MHC-II HLA-peptide complexes. Table 1 shows 32 HLA-peptide complexes which are retrieved from PDB. Note that Table 1 is the largest set of HLA-peptide complexes ever used in the literature. Each binding peptide of the 32 complexes has nine core residues (being in bold in Table 1), which are accommodated in 9 pockets labeled by P1, 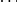, P9. We can then represent each pocket by several MHC residues, which we call “contact residues”, which are in contact with the corresponding (binding core) residue. Here for each binding core residue we define a MHC residue as a contact residue if the distance between these two residues is within 4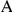 [30]. MHC sequences are retrieved from IMGT/HLA and then aligned. For each of the 32 complexes, we extract contact residues for each pocket (shown in Table S1), and then, for each pocket, use a union of contact residues over 32 complexes. Table 2 shows positions of contact residues for each pocket. We can generate a pseudosequence of each pocket for any HLA-DR allele by using a sequence of amino acids at the positions of the corresponding pocket in Table 2.

**Table 1:** Available X-ray structures of MHC class II HLA-peptide complexe.

PDB ID	HLA Allele	Peptide Sequence
1AQD	DRB1*01:01	VGSD**WRFLRGYHQ**YA
1PYW	DRB1*01:01	X**FVKQNAAAL**X
1KLG	DRB1*01:01	GEL**IGILNAAKV**PAD
2FSE	DRB1*01:01	AG**FKGEQGPKG**EPG
1KLU	DRB1*01:01	GEL**IGTLNAAKV**PAD
1SJH	DRB1*01:01	PE**VIPMFSALS**EG
1SJE	DRB1*01:01	PE**VIPMFSALS**EG
1T5W	DRB1*01:01	AA**YSDQATPLL**LSPR
1T5X	DRB1*01:01	AA**YSDQATPLL**LSPR
2IAN	DRB1*01:01	GEL**IGTLNAAKV**PAD
2IAM	DRB1*01:01	GEL**IGILNAAKV**PAD
2IPK	DRB1*01:01	XPK**WVKQNTLKL**AT
1FYT	DRB1*01:01	PK**YVKQNTLKL**AT
1R5I	DRB1*01:01	PK**YVKQNTLKL**AT
1HXY	DRB1*01:01	PK**YVKQNTLKL**AT
1JWM	DRB1*01:01	PK**YVKQNTLKL**AT
1JWS	DRB1*01:01	PK**YVKQNTLKL**AT
1JWU	DRB1*01:01	PK**YVKQNTLKL**AT
1LO5	DRB1*01:01	PK**YVKQNTLKL**AT
2ICW	DRB1*01:01	PK**YVKQNTLKL**AT
2OJE	DRB1*01:01	PK**YVKQNTLKL**AT
2G9H	DRB1*01:01	PK**YVKQNTLKL**AT
1A6A	DRB1*03:01	PVSK**MRMATPLLM**QA
1J8H	DRB1*04:01	PK**YVKQNTLKL**AT
2SEB	DRB1*04:01	AY**MRADAAAGG**A
1BX2	DRB1*15:01	ENPV**VHFFKNIVT**PR
1YMM	DRB1*15:01	ENPV**VHFFKNIVT**PRGGSGGGGG
2Q6W	DRB3*01:01	A**WRSDEALPL**GS
3C5J	DRB3*02:01	QVI**ILNHPGQI**SA
1FV1	DRB5*01:01	NPVVHF**FKNIVTPRT**PPPSQ
1H15	DRB5*01:01	GGV**YHFVKKHVH**ES
1ZGL	DRB5*01:01	VHF**FKNIVTPRT**PGG

The table shows complex structures retrieved from PDB. The columns in the table give PDB ID, HLA-DR restriction and bound peptide (binding core highlighted in bold).

**Table 2:** The HLA-DR amino acid residue positions of each pockets in TEPITOPEpan profile.

Pocket	Residue positions
P1	82 85 86 89
P2	77 78 81 82
P3	78
P4	11 13 26 28 70 71 74 78
P5	11 13 28 70 71 74
P6	11 13 28 30 61 71
P7	11 28 30 47 61 67 70 71
P8	60 61
P9	9 30 37 57 60 61

The first column gives nine pockets (P1 to P9). The second column shows corresponding residue positions in contact with each pocket.

#### Step 2: Computing the pocket similarity and weight between alleles

We compute the similarity between two pseudosequences (of query allele 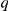 and allele 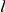 in the library of TEPITOPE) using the approach proposed in PickPocket [30]. Assuming that pocket 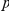 has 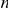 positions, for pocket 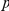, from two pseudosequence 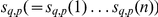 of query allele 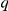 and pseudosequence 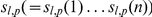 of allele 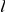, we can first compute similarity value Blosum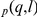 at pocket 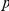 by just summing up over the similarities of all positions.
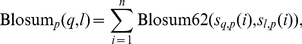
(1)where Blosum62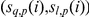 is the Blosum62 similarity score between two amino acids, 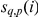 and 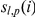. We then have the normalized similarity 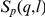 at pocket 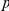 as follows:
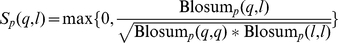
(2)where 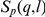 is 1 for two identical pseudosequences and 0 for totally distinctive pseudosequences. We can then compute the weight 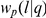 between 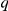 and 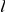 at pocket 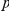, by using 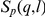 as follows:
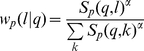
(3)where 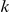 takes over 11 DRB alleles in the library of TEPITOPE, 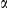 is the parameter taking a positive value only, which adjusts the contribution of alleles. A larger 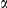 will assign lower weights to dissimilar alleles, by which similar alleles will be pronounced more.

#### Step 3: Computing PSSM

For each pocket 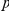, allele 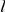 in the library has binding specificity vector 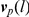 (i.e. 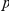-th column of PSSM). We then compute binding specificity vector 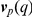 of query allele 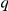 by using 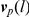 and 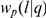 as a weighted average over all alleles in the library as follows:
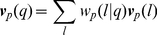
(4)


Finally we can have PSSM 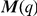 for query MHC allele 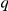 naturally as follows:
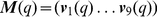
(5)In this manner, we can generate a PSSM matrix for each of over 700 HLA-DR alleles with known sequences by considering each allele as a query allele. Note that actually we used only 35 pockets (vectors) in total of 11 HLA-DR alleles, instead of all 99 ( = 9 pockets
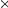
11 alleles) pockets. This is because that pockets 5 and 8 are not used in the PSSMs of TEPITOPE, and some alleles share identical binding specificities on some pockets.

## Results

We first used Nielsen-Set1 to determine the value of 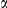 and then used the other seven datasets to compare the performance of TEPITOPEpan with those of NetMHCIIpan-2.0, NetMHCIIpan-1.0, MultiRTA and TEPITOPE. We obtained results of NetMHCIIpan-2.0 and NetMHCIIpan-1.0 by running their freely available packages and those of MultiRTA from the outputs of the web server of MultiRTA (http://bordnerlab.org/MultiRTA). We used AUC (Area under the ROC (Receiver Operator Characteristic) Curve) to measure the performance. We further used the one-tailed per-allele binomial test (excluding ties) to examine statistical significance of the performance difference between two methods, regarding those with p-values of less than 0.05 as significant cases.

### Determining 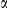


Table 3 shows the AUC of TEPITOPEpan on Nielsen-Set1 with different 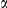, which was set from 1 to 50. Note that for each value of 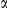, we adopted the same value for all HLA-DRB alleles. In this table, 1-KNN means the result of using only the most similar pocket specificity vector in the library of TEPITOPE. We observed that TEPITOPEpan performs better under 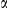 of 5 to 30, reaching the best average AUC of 0.739 in cases of 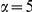 or 10. Table 3 shows that TEPITOPEpan performs better with 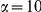 than with 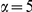 in 8 of the total 14 alleles. In addition TEPITOPEpan with 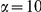 performed better than 1-KNN, being statistically significant (binomial test, *p*-value
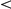
0.05) and outperformed TEPITOPE in 8 out of 11 alleles covered by TEPITOPE. We thus keep 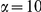 throughout all other experiments in this work.

**Table 3:** Performance of TEPITOPEpan with different alphas in terms of AUC.

Allele	Count	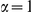	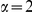	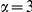	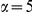	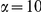	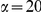	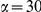	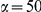	1-KNN	TEPITOPE
DRB1*01:01	1203	0.622	0.635	0.642	0.648	**0.651**	0.650	0.650	0.650	0.650	0.650
DRB1*03:01	474	0.591	0.639	0.689	0.724	**0.733**	0.729	0.728	0.728	0.729	0.727
DRB1*04:01	457	0.745	0.757	0.764	0.771	**0.773**	0.767	0.764	0.759	0.756	0.756
DRB1*04:04	168	0.819	0.832	0.836	0.842	**0.844**	0.843	0.841	0.839	0.838	0.837
DRB1*04:05	171	0.748	0.762	0.770	0.783	0.792	0.794	**0.799**	0.798	0.795	0.795
DRB1*07:01	310	0.753	0.772	**0.773**	0.770	0.767	0.767	0.767	0.767	0.766	0.766
DRB1*08:02	174	0.771	0.781	0.793	**0.797**	0.794	0.792	0.791	0.790	0.790	0.788
DRB1*09:01	117	**0.731**	0.724	0.715	0.702	0.696	0.689	0.688	0.689	0.686	0.644
DRB1*11:01	359	0.691	0.699	0.704	0.707	0.714	0.721	**0.723**	**0.723**	0.723	0.723
DRB1*13:02	179	0.725	0.735	0.744	**0.745**	0.743	0.736	0.735	0.735	0.736	0.737
DRB1*15:01	365	0.717	0.727	0.734	**0.735**	0.731	0.731	0.730	0.730	0.730	0.730
DRB3*01:01	102	0.640	0.700	0.734	**0.754**	0.731	0.707	0.700	0.663	0.606	0.673
DRB4*01:01	181	0.698	0.705	0.714	0.725	0.731	0.741	0.742	**0.743**	0.744	0.718
DRB5*01:01	343	0.626	0.638	0.644	0.645	0.651	**0.652**	0.651	0.651	0.651	0.652
Average	4603	0.706	0.722	0.732	**0.739**	**0.739**	0.737	0.737	0.733	0.729	0.728

The highest value in each row of columns for 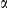 is highlighted in bold. 1-KNN means the result of using only specificity vector(s) in the library with highest similarity to derive PSSM.

### Evaluation by Nielsen-Set2

Table 4 shows the comparison result on 10 alleles in Nielsen-Set2 (which has 24 HLA-DRB alleles, but peptide data of the other 14 alleles are significantly overlapped with training data of NetMHCIIpan-1.0 and MultiRTA). This table shows that TEPITOPEpan was the second best method with an average AUC of 0.763. In fact TEPITOPEpan outperformed MultiRTA in all 10 alleles and NetMHCIIpan-1.0 in 9 out of 10 alleles, both being statistically significant (binomial test, *p*-value
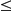
0.01). We note that the results of the best method, i.e. NetMHCIIpan-2.0, are not necessarily comparable, since exceptionally they are directly taken from [27], in which results were obtained by using LOO (Leave-one-allele-out, where the binding data of the other 23 alleles were used for training and the remaining one for testing). This means that the AUC of NetMHCIIpan-2.0 will be much lower if trained by 14 common alleles only, like NetMHCIIpan-1.0 and MutliRTA. Further note that TEPITOPEpan is robust, being unaffected by training data.

**Table 4:** AUC on Nielsen-Set2.

Allele	Count	Binder	NetMHCIIpan2.0	NetMHCIIpan-1.0	MultiRTA	TEPITOPE	TEPITOPEpan
DRB1*03:02	148	44	**0.759**	0.688	0.549		0.602
DRB1*08:06	118	91	**0.902**	0.703	0.652	0.870	0.886
DRB1*08:13	1370	455	0.666	0.763	0.712	0.746	**0.768**
DRB1*08:19	116	54	**0.813**	0.677	0.630		0.714
DRB1*12:01	117	81	0.798	0.587	0.620		**0.832**
DRB1*12:02	117	79	**0.879**	0.660	0.663		0.842
DRB1*14:02	118	78	**0.846**	0.713	0.672		0.725
DRB1*14:04	30	16	0.679	0.571	0.563		**0.683**
DRB1*14:12	116	63	**0.897**	0.797	0.688		0.804
DRB3*03:01	160	70	0.765	0.739	0.729		**0.771**
Average			0.800	0.690	0.683		0.763

The highest values for each allele are highlighted in bold. Results of NetMHCIIpan-2.0 are obtained by leave-one-(allele)-out (LOO) experiment over original 24 alleles in [27].

### Evaluation by Lin-Set3

Table 5 shows the comparison result on 7 HLA-DRB alleles in Lin-Set3. TEPITOPEpan achieved the comparable accuracy with TEPITOPE as well as NetMHCIIpan-1.0 and MultiRTA. Specifically, TEPITOPEpan outperformed TEPITOPE in 5 out of 7 alleles (with one tie), and outperformed both NetMHCIIpan-1.0 and MulitRTA in 4 out of 7 alleles.

**Table 5:** AUC on Lin-Set3.

Allele	Count	Binder	NetMHCIIpan-2.0	NetMHCIIpan-1.0	MultiRTA	TEPITOPE	TEPITOPEpan
DRB1*01:01	103	15	0.883	0.846	0.817	**0.892**	**0.892**
DRB1*03:01	103	18	0.716	0.668	**0.757**	0.695	0.680
DRB1*04:01	103	8	**0.845**	0.814	0.696	0.754	0.782
DRB1*07:01	103	10	**0.878**	0.852	0.781	0.740	0.741
DRB1*11:01	103	39	**0.883**	0.820	0.819	0.824	0.826
DRB1*13:01	103	11	**0.728**	0.715	0.686	0.715	0.716
DRB1*15:01	103	11	**0.838**	0.790	0.689	0.659	0.661
Average			0.824	0.786	0.749	0.754	0.757

The highest value for each allele is highlighted in bold. According to Nielsen et al. [27], for DRB1*01:01, 04:01, 07:01 and 15:01, binding threshold is set to 100 nM, and threshold is set to 1000 nM for the rest when calculating the AUC.

### Evaluation by Epan-Set4

Table 6 shows the comparison result on Epan-Set4, which is our original, well-qualified dataset. Again TEPITOPEpan performed the second best, next to NetMHCIIpan-2.0, on average, and outperformed TEPITOPE, MultiRTA and NetMHCIIpan-1.0. Concretely, out of 14 alleles, TEPITOPEpan outperformed MultiRTA in 9, NetMHCIIpan-1.0 in 7, and NetMHCIIpan- 2.0 in 6 alleles, which are all in 12 alleles not covered by Nielsen-Set2.

**Table 6:** AUC on Epan-Set4.

Allele	Count	Binder	NetMHCIIpan-2.0	NetMHCIIpan-1.0	MulitRTA	TEPITOPE	TEPITOPEpan
DRB1*01:02	92	62	0.746	**0.785**	0.749	0.762	0.758
DRB1*01:03	52	41	0.772	0.756	0.772		**0.867**
DRB1*03:02	88	44	**0.840**	0.775	0.733		0.823
DRB1*04:03	63	14	0.678	0.659	0.611		**0.762**
DRB1*04:06	92	37	0.486	**0.557**	0.519		0.501
DRB1*11:02	65	30	**0.774**	0.738	0.591	0.723	0.738
DRB1*11:03	64	27	**0.791**	0.623	0.585		0.726
DRB1*11:04	73	34	**0.737**	0.639	0.618	0.664	0.654
DRB1*12:01	719	446	**0.740**	0.721	0.673		0.659
DRB1*13:01	302	132	0.494	0.516	0.567	**0.637**	0.623
DRB1*14:01	43	33	0.676	0.761	**0.809**		0.785
DRB1*15:02	47	21	**0.888**	0.762	0.777	0.740	0.742
DRB1*16:01	56	17	**0.814**	0.793	0.789		0.644
DRB3*02:02	656	318	**0.806**	0.732	0.680		0.686
Average			**0.732**	0.701	0.677		0.712
Average (Tepitope alleles)			**0.728**	0.688	0.661	0.705	0.703
Average (Others)			**0.734**	0.708	0.686		0.717

Highest values for each allele are highlighted in bold.

### Evaluation by Bordner-Set5

The comparison result on Bordner-Set5 (HLA-DRB1*13:01) shows that TEPITOPEpan achieved the highest AUC value of 0.833, being followed by MutliRTA (AUC of 0.761), NetMHCIIpan-1.0 (AUC of 0.719) and NetMHCIIpan-2.0 (AUC of 0.690).

### Evaluation by Peptide Binding Motifs

**Figure 1:**
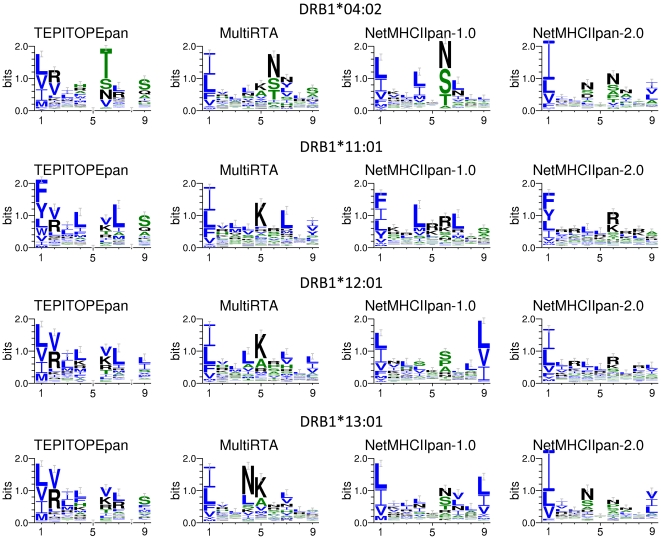
Comparing of different pan-specific methods by the sequence logos of peptides restricted to HLA-DRB1*04:02, DRB1*11:01, DRB1*12:01, DRB1*13:01.

We explored the difference of four competing methods: TEPITOPEpan, MultiRTA, NetMHCIIpan-1.0 and NetMHCIIpan-2.0, by visualizing binding motifs of alleles as sequence logos [33], using the following procedure: We first generated 100,000 peptides from SWISS-PROT randomly. Then, for each of the four methods, we computed binding scores of the 100,000 peptides and selected the top 1% peptides in scores to draw sequence logos, as described in [14]. Figure 1 shows the sequence logos (binding motifs) of the four methods on four alleles DRB1*04:02, DRB1*11:01, DRB1*12:01 and DRB1*13:01 obtained in this manner (see Figure S1 and S2 for more sequence logos of other 12 HLA-DR alleles). In Figure 1, the height in a column indicates the relative information content of the corresponding pocket in the motif, and the height of each letter stands for the amino acid frequency in the corresponding pocket. TEPITOPEpan has already achieved a good performance on these four alleles. In addition, the sequence logos by TEPITOPEpan were consistent with known binding motifs in SYFPEITHI [32] and the HLA FactsBook [34]. We first note that all methods agreed that the information content of the first pocket is high and amino acids preferred in this pocket are relatively fixed, indicating that the first pocket, i.e. P1, must be a *primary anchor*. In addition, most methods suggested that P4, P6, P7 and P9 are primary anchors. Meanwhile, we could observe obvious differences in primary anchors among sequence logos by these four methods. For example, P5 was identified as a primary anchor by MultiRTA, while P5 and P8 were not thought as primary anchors in TEPITOPEpan at all. More specifically, in SYFPEITHI, the motif of DRB1*04:02 is [VILM]xx[YFWILMRN]x[NQSTK][RKHNQP]x[DEHLNQ RSTYCILMVHA]. The sequence logos of most methods were consistent with this motif at major primary anchors, such as P1 and P6, while amino acids by competing methods were very different from each other at P4. At P4, TEPITOPEpan suggested arginine (R) and phenylalanine (F), which were the most consistent with the motif in SYFPEITHI. Similarly, the binding motif of DRB1*1301 in SYFPEITHI is [ILV]xx[LVMAWY]x[RK]xx[YFAST], and [IVF]xx[YWLVAM]x[RK]xx[YFAST] is reported in [34]. Both of them indicate that lysine (K) and arginine (R) are at P6, which, as indicated by Figure 1, were captured by TEPITOPEpan only. For DRB1*11:01, sequence logos by TEPITOPEpan were relatively similar to those of NetMHCIIpan-1.0 and NetMHCIIpan-2.0, particularly leucine (L) at P4, arginine (R) and lysine (K) at P6 and serine (S) and alanine (A) at P9, which were all consistent with the binding motif in SYFPEITHI. Finally for DRB1*1201, a worth mentioning result is that only TEPITOPEpan suggested valine (V) at P6, which is clearly consistent with the binding motif reported in SYFPEITHI database. Overall the sequence logos showed unique characteristics of each competing method, helping to understand the binding specificities of various MHC alleles. For the four alleles, the sequence logos by TEPITOPEpan demonstrated that they are consistent with known binding motifs the most.

### Evaluation by SYF-Set6 and EIEDB-Set7: Identifying HLA-DR ligands and T-cell epitopes

Table 7 summarizes the results of comparison experiment on SYF-Set6 for identifying HLA-DR ligands and EIEDB-Set7 for detecting T-cell epitopes. For SYF-Set6, AUC was in the order of NetMHCIIpan-2.0, TEPITOPEpan, NetMHCIIpan-1.0, and MultiRTA for Avg per ligand, while for Avg per allele, the order of AUC was NetMHCIIpan-2.0, NetMHCIIpan-1.0, TEPITOPEpan and MultiRTA. Interestingly, for EIEDB-Set7, the order of Avg per epitope was the same as that of Avg per allele in SYF-Set6, and that of Avg per allele was the same as that of Avg per ligand in SYF-Set6. This indicates that the AUC of TEPITOPEpan was comparable against NetMHCIIpan-1.0, while these two methods outperformed MultiRTA in EIEDB-Set7 (both per allele and per epitope) and SYS-Set6 (per ligand), being statistically significant (binomial test, *p*-value 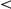
0.05). Similarly note that NetMHCIIpan-2.0 outperformed these three methods on both datasets, being statistically significant (binomial test, *p*-value 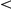
0.05), except NetMHCIIpan-1.0 on SYF-Set6. Thus we can say that TEPITOPEpan is the second best method in identifying HLA-DR ligands and T-cell epitopes.

**Table 7:** Evaluation on SYF-Set6 and EIEDB-SET7.

SYF-Set6	Ligand	NetMHCIIpan-2.0	NetMHCIIpan-1.0	MultiRTA	TEPITOPE	TEPITOPEpan
Avg per ligand	1164	**0.829**	0.799	0.760		0.800
Avg per allele	28	**0.797**	0.787	0.756		0.769
Avg per allele (TEPITOPE alleles)	17	0.785	0.767	0.733	**0.811**	0.807
Avg per allele (Other alleles)	11	0.814	**0.818**	0.791		0.711

EIEDB-Set7	Epitope	NetMHCIIpan-2.0	NetMHCIIpan-1.0	MultiRTA	TEPITOPE	TEPITOPEpan
Avg per epitope	1325	**0.751**	0.729	0.696		0.725
Avg per allele	42	**0.781**	0.759	0.717		0.762
Avg per allele (TEPITOPE alleles)	20	0.747	0.744	0.696	**0.757**	0.755
Avg per allele (Other alleles)	22	**0.811**	0.772	0.736		0.769

Identifying HLA-DR ligands and T-cell epitopes, respectively. Ligand and Epitopes show the number of HLA-DR ligands and HLA-DR epitopes, respectively. Avg per ligand shows the average AUC over all ligands, and Avg per allele gives the average of Avg per ligand over all alleles.

For 20 alleles used by TEPITOPE, on both datasets, there were no significant differences among TEPITOPEpan, NetMHCIIpan-1.0, NetMHCIIpan-2.0 and TEPITOPE, all having outperformed MultiRTA on at least 14 alleles. On the other hand, for other alleles, the AUC of TEPITOPEpan was lowered, and even MultiRTA outperformed TEPITOPEpan for 9 out of 11 alleles in SYF-Set6, being statistically significant (binomial test, p-value
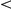
0.05). Detailed results are shown in Table S2 and S3.

### Evaluation by EpanCore-Set8: Identifying the binding core

Table 8 shows the number of errors in predicting the binding core of 20 known 3D complex structures in EpanCore-Set8. On predicting the position of the binding core in a given sequence, if the position was incorrect (not exact), we counted that prediction as an error. This table shows that the number of errors by TEPITOPEpan was the smallest among 4 competing methods that can cover all HLA-DR alleles. We emphasize that TEPITOPEpan achieved the smallest number, being better than even NETMHCIIpan-2.0, implying that the predictive power of TEPITOEpan would be comparable or might be better against NETMHCIIpan-2.0 under the setting of not only giving a score to a query peptide but also predicting the binding core exactly. Detailed results are shown in Table S4.

**Table 8:** The number of errors on predicting binding cores of 20 complexes in EpanCore-Set8.

PDB	#complexes	#alleles	NetMHCIIpan-2.0	NetMHCIIpan-1.0	MultiRTA	TEPITOPEpan	TEPITOPE
Count	20	7	5 errors	3 errors	3 errors	2 error	0 errors (2 missing)

The binding cores of 2 complexes cannot be predicted by TEPITOPE, since it doesn't cover DRB3*01:01 and DRB3*02:01.

## Discussion

We have presented TEPITOPEpan that extends TEPITOPE to predicting over 700 HLA-DR alleles with known sequences. Note that TEPITOPEpan is simple, because of its PSSM-based nature. Extensive experiments were conducted on a variety of datasets to validate its performance. The summary of results showed that, among the four methods in the benchmark, TEPITOPEpan achieved roughly the second best performance in predicting binding peptides of novel HLA-DR molecules and identifying HLA-DR ligand and T-cell epitopes. In addition, TEPITOPEpan achieved the best performance on identifying the bind core of a given peptide. The most notable point is that TEPITOPEpan does not need a large amount of training data which are usually required for the other machine learning-based methods, but only the sequence of a target allele.

The state-of-the-art pan-specific methods are generally based on machine learning, requiring plenty of quantitative binding data. Our experimental results demonstrate that their performances on novel alleles do not necessarily reach a satisfactory level. It suggests that trained models cannot be easily generalized to other alleles. This may be due to several reasons. First, the design principles of some pan-specific methods are oversimplified by which the binding mechanism between MHC molecules and peptides cannot be fully captured. Second, some pan-specific methods may be overfitted to training alleles, and thus cannot achieve good performance on novel alleles. Third, high-quality experimental data are not enough for building an accurate pan-specific method. Fourth, binding specificities of some novel alleles might be different from those used for training the model. All these points imply that improving existing pan-specific methods or developing a more accurate method is vital for boosting the accuracy of pan-specific MHC-II binding peptide prediction.

TEPITOPEpan was based on 35 unique binding specificity vectors in TEPITOPE, which were obtained by biological experiments on 11 HLA-DR alleles. Although the performance of TEPITOPEpan has validated the reasonability of these vectors in this paper, we still have to say that only 35 vectors are insufficient. In fact, thousands of MHC class II molecules were sequenced and many MHC-peptide complex structures have been experimentally determined. A natural idea is to use the binding specificities of new pockets of these MHC molecules by measuring them using the state-of-the-art biochemical experiments. This would make TEPITOPEpan achieve a higher performance. Another possible direction would be to extend peptide specificity prediction to other MHC-II alleles like HLA-DP, -DQ alleles and even MHC-II alleles of non-human species.

## Supporting Information

Figure S1 Comparing of different pan-specific methods by the sequence logos of peptides restricted to HLA-DRB1*01:02, DRB1*01:03, DRB1*03:02, DRB1*04:03, DRB1*04:04, DRB1*04:05.
(PDF)

Figure S2 Comparing of different pan-specific methods by the sequence logos based on sampled binding peptides restricted to HLA-DRB1*08:13, HLA-DRB1*11:02, DRB1*11:03, DRB1*11:04, DRB1*14:01, DRB1*14:04.
(PDF)

Table S1 **Composing residues of each pocket extracted from 32 complex structures.** **The** first column gives PDB IDs of 32 MHC-II HLA-peptide complex structures from PDB. The next 9 columns give extracted composing residues of nine pockets of the HLA-DR molecule in the corresponding complex, respectively. Each element,e.g. 82 N, consists of an index number and the residue on that site. The last row gives a union set of composing residue indexes.
(PDF)

Table S2 **Evaluation of different methods on identifying endogenous HLA-DR ligands from SYFPEITHI database.** Elements in the table are values of AUC and largest value of each row is highlighted in bold. Predictions of NetMHCIIpan-1.0 and 2.0 were obtained from their stand-alone packages. Predictions of MultiRTA were from its web server. Count gives the number of HLA-DR ligands retrieved from SYFPEITHI. Ave per ligand gives the average AUC over all 1164 ligands. Ave per allele gives the average of per-ligand-average AUCs of all alleles.
(PDF)

Table S3 **Evaluation of different methods on identifying HLA-DR T cell epitopes retrieved from IEDB.** Elements in the table are values of AUC and largest value of each row is highlighted in bold. Predictions of NetMHCIIpan-1.0 and 2.0 were obtained from their standalone packages. Predictions of MultiRTA were from its web server. Count gives the number of HLA-DR epitopes retrieved from IEDB. Ave per epitope gives the average AUC over all 1325 epitopes. Ave per allele gives an average of per-epitope-average AUCs of all alleles.
(PDF)

Table S4 **Evaluation on identifying binding core.** The table shows complexes with known binding cores retrieved from PDB. The first two columns in the table give PDB ID, HLA-DR restriction, bound peptide and experimentally determined binding core, respectively. Twenty distinct structures in terms of allele and peptide sequence are labeled with an asterisk. The last columns give predicted cores of different methods. Predictions of different methods were obtained from their stand-alone packages or web servers. Prediction results based on 20 distinct structures are shown in brackets with an asterisk. Additionally, TEPITOPE can not make prediction for DRB3*01:01 and DRB3*02:01.
(PDF)
