# Supplementary material for: TEPITOPEpan: Extending TEPITOPE for Peptide Binding Prediction Covering over 700 HLA-DR Molecules
Source: PLoS One. 2012 Feb 23;7(2):e30483. doi: 10.1371/journal.pone.0030483 (PMC3285624; doi:10.1371/journal.pone.0030483)
Supplement: Figure S1 — Comparing of different pan-specific methods by the sequence logos of peptides restricted to HLA-DRB1*01:02, DRB1*01:03, DRB1*03:02, DRB1*04:03, DRB1*04:04, DRB1*04:05. (PDF) [file pone.0030483.s001.pdf]

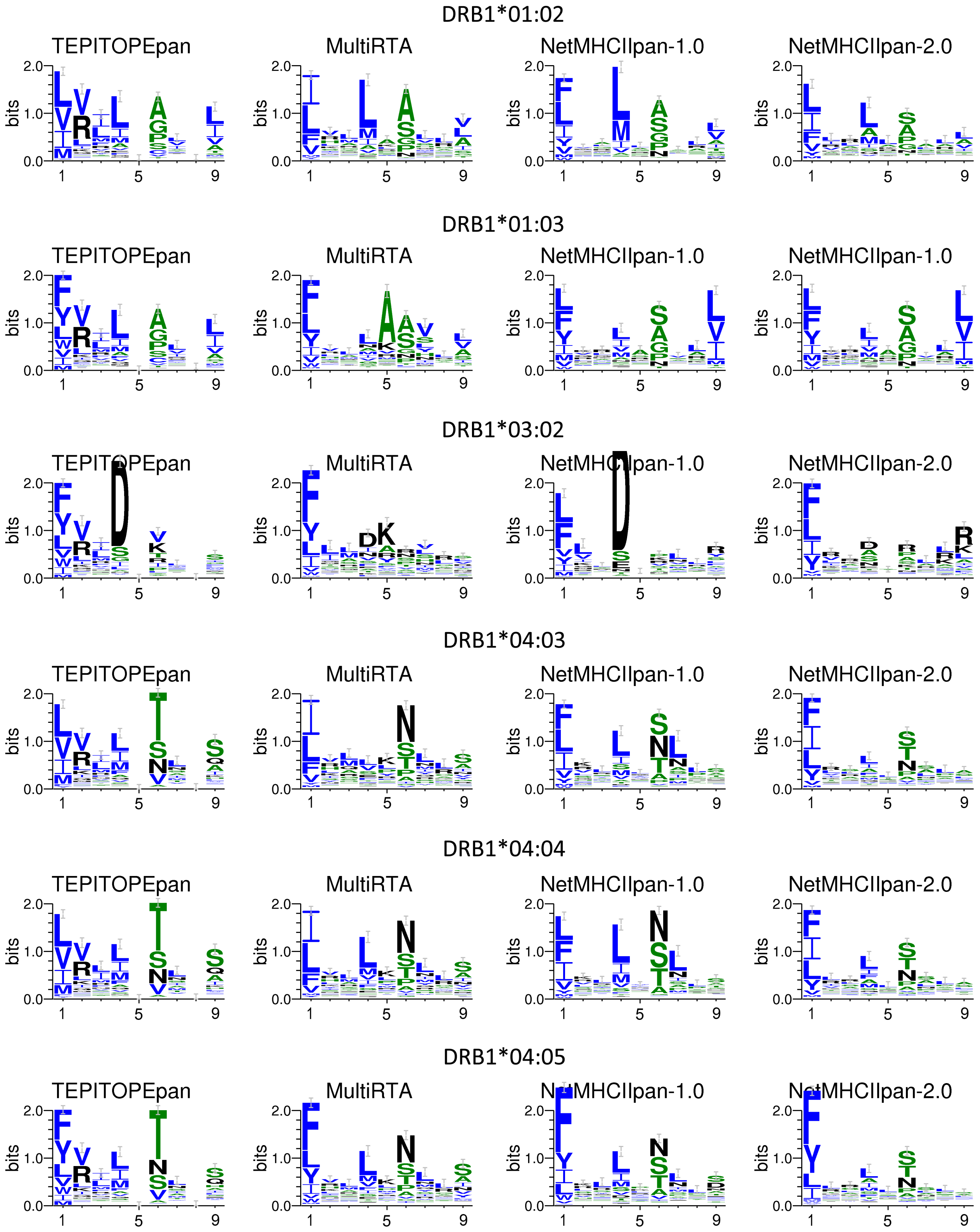

Figure S1: Comparing of different pan-specific methods by the sequence logos of peptides restricted to HLA-DRB1*01:02, DRB1*01:03, DRB1*03:02, DRB1*04:03, DRB1*04:04, DRB1*04:05.
