# Supplementary material for: TEPITOPEpan: Extending TEPITOPE for Peptide Binding Prediction Covering over 700 HLA-DR Molecules
Source: PLoS One. 2012 Feb 23;7(2):e30483. doi: 10.1371/journal.pone.0030483 (PMC3285624; doi:10.1371/journal.pone.0030483)
Supplement: Figure S2 — Comparing of different pan-specific methods by the sequence logos based on sampled binding peptides restricted to HLA-DRB1*08:13, HLA-DRB1*11:02, DRB1*11:03, DRB1*11:04, DRB1*14:01, DRB1*14:04. (PDF) [file pone.0030483.s002.pdf]

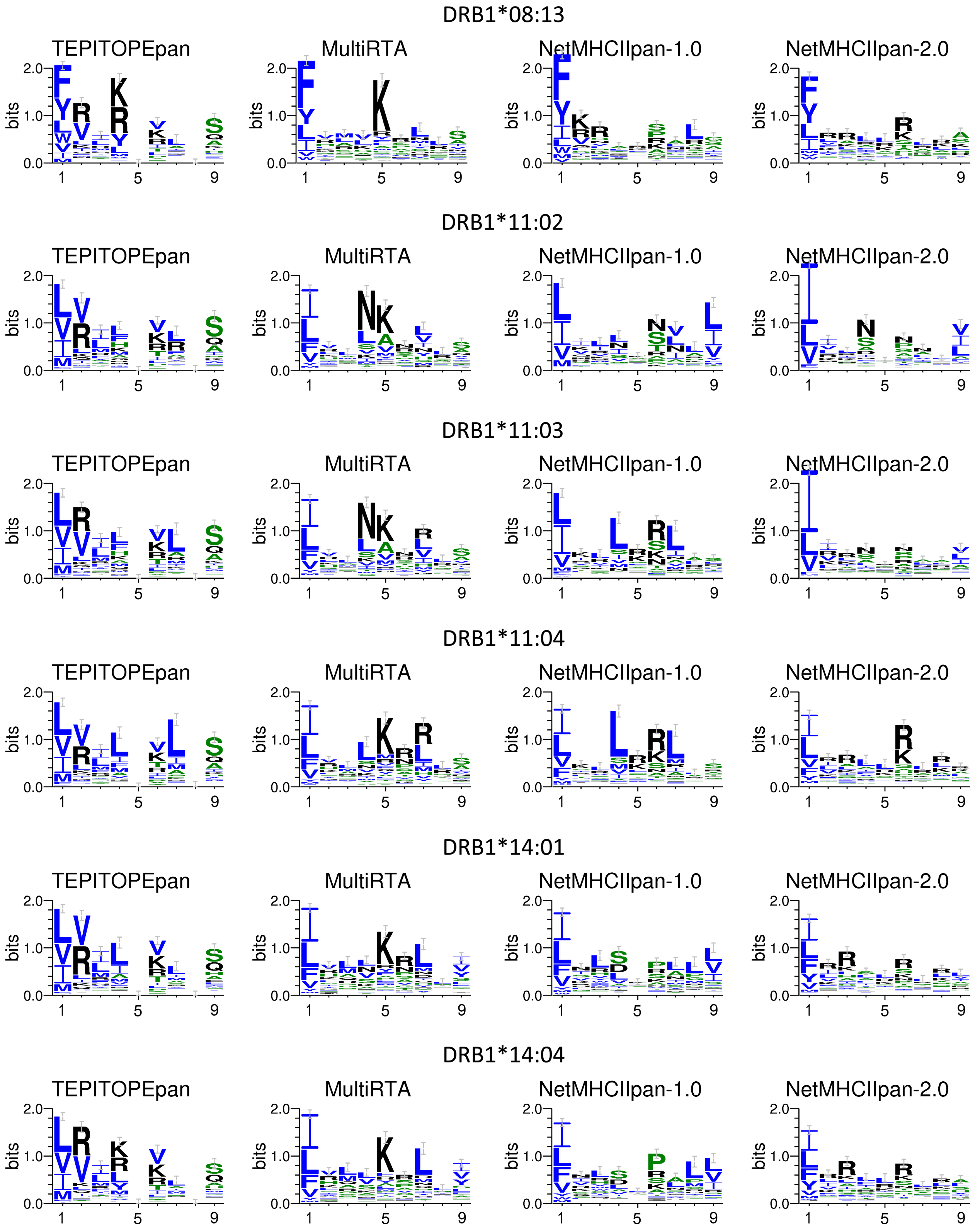

Figure S2: Comparing of different pan-specific methods by the sequence logos based on sampled binding peptides restricted to HLA-DRB1*08:13, DRB1*11:02, DRB1*11:03, DRB1*11:04, DRB1*14:01, DRB1*14:04.
