# Supplementary material for: TEPITOPEpan: Extending TEPITOPE for Peptide Binding Prediction Covering over 700 HLA-DR Molecules
Source: PLoS One. 2012 Feb 23;7(2):e30483. doi: 10.1371/journal.pone.0030483 (PMC3285624; doi:10.1371/journal.pone.0030483)
Supplement: Table S1 — Composing residues of each pocket extracted from 32 complex structures. The first column gives PDB IDs of 32 MHC-II HLA-peptide complex structures from PDB. The next 9 columns give extracted composing residues of nine pockets of the HLA-DR molecule in the corresponding complex, respectively. Each element,e.g. 82 N, consists of an index number and the residue on that site. The last row gives a union set of composing residue indexes. (PDF) [file pone.0030483.s003.pdf]

Table S1: Composing residues of each pocket extracted from 32 complex structures. The first column gives PDB IDs of 32 MHC-II HLA-peptide complex structures from PDB. The next 9 columns give extracted composing residues of nine pockets of the HLA-DR molecule in the corresponding complex, respectively. Each element,e.g. 82N, consists of an index number and the residue on that site. The last row gives a union set of composing residue indexes.

PDB	Pocket1	Pocket2	Pocket3	Pocket4	Pocket5	Pocket6	Pocket7	Pocket8	Pocket9
1AQD	82N 85V 86G	77T 78Y 81H 82N	78Y	13F 74 A 78Y	13F 71R	11L	47Y 61W 67L 70 Q 71R	60Y 61W	9W 57D 61W
1PYW	82N 85V 86G 89F	77T 78Y 81H 82N	78Y	13F 70Q 71R 74A 78Y	13F 71R	11L	11L 28E 61W 71R	60Y 61W	57D 61W
1KLG	82N 85V	78Y 81H 82N	78Y	13F 71R 78Y	13F 71R	11L	61W	60Y 61W	57D 61W
2FES	82N 85V 86G 89F	77T 78Y 82N		13F 28E 70Q 71R 74A 78Y	13F 71R	71R	28E 47Y 61W 67L 71R	61W	57D
1KLU	82N 85V	78Y 81H 82N		13F 71R 78Y	13F 71R	11L	61W	60Y 61W	57D 61W
1SJH	82N	78Y 81H 82N		13F 26L 70Q 71R 74A 78Y	71R	11L	61W	60Y 61W	57D 61W
1SJE	82N	78Y 81H 82N	78Y	13F 26L 70Q 71R 74A 78Y	71R	11L	61W	60Y 61W	57D 60Y 61W
1T5W	82N 86G 89F	78Y 81H 82N	78Y	13F 70Q 71R 74A 78Y	13F 71R	11L	61W 71R	60Y 61W	9W 57D 61W
1T5X	82N 86G 89F	78Y 81H 82N	78Y	13F 70Q 71R 74A 78Y	71R	11L	61W 71R	61W	57D 61W
2IAN	82N 85V	78Y 81H 82N	78Y	13F 70Q 74A 78Y	13F 70Q 71R	11L	61W 71R	61W	57D 61W
2IPK	82N 85V 86G 89F	77T 78Y 81H 82N		13F 70Q 71R 74A 78Y	71R	11L	47Y 61W 67L 71R	60Y 61W	9W 57D 61W
1FYT	82N 85V 86G 89F	78Y 81H 82N	78Y	13F 70Q 71R 74A 78Y	13F 71R	11L	28E 47Y 61W 67L 71R	60Y 61W	9W 57D 61W
1R5I	82N 85V 86G 89F	77T 78Y 81H 82N	78Y	13F 70Q 71R 74A 78Y	70Q 71R	11L	47Y 61W 67L 71R	61W	9W 57D 61W
1HXY	82N 85V 86G 89F	78Y 81H 82N		13F 70Q 71R 74A 78Y	71R	11L	28E 47Y 61W 67L 71R	60Y 61W	9W 57D 61W
1JWM	82N 85V 86G 89F	78Y 81H 82N	78Y	13F 70Q 71R 74A 78Y	71R	11L	28E 47Y 61W 67L 71R	61W	57D 61W
1JWS	82N 85V 86G 89F	78Y 81H 82N	78Y	13F 70Q 71R 74A 78Y	13F 71R	11L	47Y 61W 67L 71R	61W	9W 57D 61W
1JWU	82N 85V 86G 89F	78Y 81H 82N	78Y	13F 70Q 71R 74A 78Y	13F 71R	11L	28E 47Y 61W 67L 71R	61W	9W 57D 61W
1LO5	82N 85V 86G 89F	78Y 81H 82N	78Y	13F 70Q 78Y	13F 71R	11L	47Y 61W 67L 71R	61W	9W 57D 60Y 61W
2ICW	82N 85V 86G 89F	78Y 81H 82N	78Y	13F 70Q 71R 74A 78Y	13F 71R	11L	28E 47Y 61W 67L 71R	61W	9W 57D 61W
2OJE	82N 85V 86G	77T 78Y 81H 82N	78Y	13F 70Q 71R 74A 78Y	70Q 71R	11L	28E 47Y 61W 67L 71R	61W	9W 57D 61W
2G9H	82N 85V 86G 89F	77T 78Y 81H 82N	78Y	13F 70Q 71R 74A 78Y	71R	11L 13F	28E 47Y 61W 67L 71R	60Y 61W	9W 57D 61W
2IAM	82N	78Y 81H 82N	78Y	13F 70Q 71R 74A 78Y	70Q 71R	11L	61W 67L 71R	60Y 61W	57D 61W
1A6A	82N 85V 86V	77N 78Y 81H 82N	78Y	13S 26Y 74R 78Y	71K 74R	11S 30Y	30Y 47F 61W 67L 71K	61W	9E 30Y 57D 61W
1J8H	82N 85V 86G 89F	77T 78Y 81H 82N	78Y	13H 26F 28D 70Q 71K 78Y	13H 70Q 71K	11V 13H 30Y	30Y 47Y 61W 67L	60Y 61W	37Y 57D 61W
2SEB	82N	77T 78Y 81H 82N		13H 26F 71K 78Y	13H 71K	30Y	30Y 47Y 61W	60Y 61W	61W
1BX2	82N 85V	77T 78Y 81H 82N	78Y	13R 26F 28D 70Q 74A 78Y	70Q	13R			57D 60Y 61W
1YMM	82N	77T 78Y 81H 82N	78Y	13R 26F 28D 70Q 71A 78Y	70Q	13R	61W 67I	61W	57D 61W
2Q6W	82N 85V 86G	77N 78Y 81H 82N	78Y	11R 13S 26Y 28D 71K 74R 78Y	11R 70Q 71K	11R	30Y 47Y 61W 67L 71K	61W	37F 57V 61W
3C5J	82N 85V 86V	77N 78Y 81H 82N	78Y	13S 26F 28E 71K 74Q 78Y	28E 71K	11L 30Y	30Y 61W	61W	61W
1FV1	82N 85V 86G 89F	78Y 81H 82N	78Y	13Y 71R 78Y	71R	13Y	61W 67F 71R	61W	57D
1H15	82N 89F	77T 78Y 81H 82N	78Y	13Y 71R 78Y	71R	11D 13Y 30D	61W		57D 60Y
1ZGL	82N 85V 89F	77T 78Y 81H 82N		13Y 26F 71R 78Y	13Y	13Y 28H 61W 71R	61W		57D 60Y 61W
Union	82 85 86 89	77 78 81 82	78	11 13 26 28 70 71 74 78	11 13 28 70 71 74	11 13 28 30 61 71	11 28 30 47 61 67 70 71	60 61	9 30 37 57 60 61
