# Supplementary material for: TEPITOPEpan: Extending TEPITOPE for Peptide Binding Prediction Covering over 700 HLA-DR Molecules
Source: PLoS One. 2012 Feb 23;7(2):e30483. doi: 10.1371/journal.pone.0030483 (PMC3285624; doi:10.1371/journal.pone.0030483)
Supplement: Table S2 — Evaluation of different methods on identifying endogenous HLA-DR ligands from SYFPEITHI database. Elements in the table are values of AUC and largest value of each row is highlighted in bold. Predictions of NetMHCIIpan-1.0 and 2.0 were obtained from their stand-alone packages. Predictions of MultiRTA were from its web server. Count gives the number of HLA-DR ligands retrieved from SYFPEITHI. Ave per ligand gives the average AUC over all 1164 ligands. Ave per allele gives the average of per-ligand-average AUCs of all alleles. (PDF) [file pone.0030483.s004.pdf]

Table S2: Evaluation of different methods on identifying endogenous HLA-DR ligands from SYFPEITHI database. Elements in the table are values of AUC and largest value of each row is highlighted in bold. Predictions of NetMHCIIpan-1.0 and 2.0 were obtained from their stand-alone packages. Predictions of MultiRTA were from its web server. Count gives the number of HLA-DR ligands retrieved from SYFPEITHI. Ave per ligand gives the average AUC over all 1164 ligands. Ave per allele gives the average of per-ligand-average AUCs of all alleles.

Allele	Count	NetMHCpan-2.0	NetMHCpan-1.0	MultiRTA	TEPITOPE	TEPITOPEpan
HLA-DRB1*01:01	53	0.835	0.827	0.834	**0.846**	**0.846**
HLA-DRB1*01:02	5	0.927	0.878	**0.934**	0.886	0.899
HLA-DRB1*03:01	88	**0.789**	0.710	0.651	0.692	0.688
HLA-DRB1*04:01	468	**0.875**	0.823	0.770	0.844	0.837
HLA-DRB1*04:02	36	0.666	0.723	0.768	**0.889**	0.884
HLA-DRB1*04:03	1	0.845	0.991	**1.000**		0.954
HLA-DRB1*04:04	42	0.764	0.793	0.722	0.790	**0.810**
HLA-DRB1*04:05	36	**0.855**	0.850	0.729	0.832	0.806
HLA-DRB1*07:01	47	**0.744**	0.707	0.720	0.708	0.707
HLA-DRB1*08:01	39	0.642	0.655	0.541	**0.721**	0.682
HLA-DRB1*08:02	1	0.978	**0.982**	0.532	0.916	0.926
HLA-DRB1*08:03	1	0.292	**0.659**	0.383		0.164
HLA-DRB1*09:01	6	**0.957**	0.875	0.842		0.673
HLA-DRB1*10:01	183	0.866	**0.869**	0.826		0.787
HLA-DRB1*11:01	35	**0.896**	0.847	0.838	0.847	0.843
HLA-DRB1*11:04	8	0.911	**0.918**	0.812	0.886	0.876
HLA-DRB1*12:01	11	**0.863**	0.818	0.847		0.834
HLA-DRB1*13:01	16	0.724	0.682	0.744	**0.835**	0.823
HLA-DRB1*13:02	19	0.559	0.550	0.721	**0.750**	0.744
HLA-DRB1*14:01	9	**0.809**	0.717	0.733		0.727
HLA-DRB1*15:01	22	0.671	0.698	0.663	**0.728**	0.726
HLA-DRB1*15:02	3	0.666	0.568	0.705	0.779	**0.786**
HLA-DRB1*16:01	2	0.846	**0.941**	0.915		0.641
HLA-DRB3*01:01	2	**0.971**	0.957	0.952		0.942
HLA-DRB3*03:01	5	**0.948**	0.827	0.939		0.782
HLA-DRB4*01:01	6	**0.729**	0.554	0.515		0.556
HLA-DRB4*01:03	2	**0.827**	0.793	0.744		0.762
HLA-DRB5*01:01	18	**0.847**	0.825	0.777	0.832	0.830
Ave per ligand	1164	**0.829**	0.799	0.760		0.800
Ave per allele	28	**0.797**	0.787	0.756		0.769
Tepitope	17	0.785	0.767	0.733	**0.811**	0.807
!Tepitope	11	0.814	**0.818**	0.791		0.711
