# Supplementary material for: TEPITOPEpan: Extending TEPITOPE for Peptide Binding Prediction Covering over 700 HLA-DR Molecules
Source: PLoS One. 2012 Feb 23;7(2):e30483. doi: 10.1371/journal.pone.0030483 (PMC3285624; doi:10.1371/journal.pone.0030483)
Supplement: Table S3 — Evaluation of different methods on identifying HLA-DR T cell epitopes retrieved from IEDB. Elements in the table are values of AUC and largest value of each row is highlighted in bold. Predictions of NetMHCIIpan-1.0 and 2.0 were obtained from their standalone packages. Predictions of MultiRTA were from its web server. Count gives the number of HLA-DR epitopes retrieved from IEDB. Ave per epitope gives the average AUC over all 1325 epitopes. Ave per allele gives an average of per-epitope-average AUCs of all alleles. (PDF) [file pone.0030483.s005.pdf]

Table S3: Evaluation of different methods on identifying HLA-DR T cell epitopes retrieved from IEDB. Elements in the table are values of AUC and largest value of each row is highlighted in bold. Predictions of NetMHCIIpan-1.0 and 2.0 were obtained from their standalone packages. Predictions of MultiRTA were from its web server. Count gives the number of HLA-DR epitopes retrieved from IEDB. Ave per epitope gives the average AUC over all 1325 epitopes. Ave per allele gives an average of per-epitope-average AUCs of all alleles.

Allele	Count	NetMHCIIpan-2.0	NetMHCIIpan-1.0	MultiRTA	TEPITOPE	TEPITOPEpan
HLA-DRB1*01:01	125	**0.808**	0.774	0.786	0.803	0.803
HLA-DRB1*01:02	4	**0.879**	0.852	0.823	0.846	0.796
HLA-DRB1*01:03	5	0.666	**0.754**	0.528		0.736
HLA-DRB1*03:01	173	**0.683**	0.673	0.655	0.631	0.633
HLA-DRB1*04:01	342	**0.775**	0.757	0.707	0.757	0.752
HLA-DRB1*04:02	33	0.570	0.545	0.521	0.575	**0.576**
HLA-DRB1*04:03	14	**0.895**	0.888	0.848		0.864
HLA-DRB1*04:04	46	0.744	0.743	0.715	0.743	**0.745**
HLA-DRB1*04:05	21	0.625	0.641	0.579	0.752	**0.727**
HLA-DRB1*04:06	6	0.739	0.793	**0.869**		0.782
HLA-DRB1*04:07	4	0.671	0.792	0.749		**0.847**
HLA-DRB1*04:08	2	0.986	0.979	**1.000**	0.927	0.927
HLA-DRB1*07:01	56	0.742	**0.760**	0.736	0.742	0.743
HLA-DRB1*07:03	1	0.896	0.913	0.707	**0.914**	**0.914**
HLA-DRB1*08:01	4	0.663	0.655	**0.716**	0.620	0.655
HLA-DRB1*08:02	2	0.754	**0.940**	0.685	0.833	0.856
HLA-DRB1*08:03	2	0.852	**0.918**	0.707		0.585
HLA-DRB1*09:01	13	**0.738**	0.610	0.636		0.732
HLA-DRB1*10:01	4	**0.875**	0.744	0.790		0.854
HLA-DRB1*11:01	88	**0.815**	0.774	0.703	0.769	0.763
HLA-DRB1*11:02	1	0.495	0.662	0.503	**0.876**	0.851
HLA-DRB1*11:03	3	0.510	**0.680**	0.480		0.336
HLA-DRB1*11:04	6	0.807	0.728	0.668	0.790	**0.811**
HLA-DRB1*12:01	3	**0.970**	0.749	0.862		0.874
HLA-DRB1*13:01	15	0.631	0.621	0.642	**0.775**	0.762
HLA-DRB1*13:02	10	**0.859**	0.722	0.790	0.756	0.751
HLA-DRB1*13:03	3	0.603	**0.719**	0.515		0.584
HLA-DRB1*14:01	16	**0.788**	0.730	0.696		0.733
HLA-DRB1*14:04	1	0.955	**0.986**	0.938		0.948
HLA-DRB1*14:05	2	0.839	**0.871**	0.848		0.807
HLA-DRB1*15:01	193	**0.722**	0.714	0.665	0.692	0.692
HLA-DRB1*15:02	20	**0.681**	0.659	0.570	0.587	0.591
HLA-DRB1*15:03	2	**0.874**	0.707	0.531		0.835
HLA-DRB1*16:01	5	0.723	**0.779**	0.721		0.677
HLA-DRB1*16:02	3	**0.984**	0.912	0.885		0.929
HLA-DRB3*01:01	12	**0.895**	0.894	0.882		0.845
HLA-DRB3*02:02	10	0.539	0.423	0.466		**0.620**
HLA-DRB3*03:01	1	**0.966**	0.891	0.906		0.910
HLA-DRB4*01:01	17	**0.787**	0.471	0.583		0.585
HLA-DRB4*01:03	1	**0.991**	0.946	**0.991**		0.990
HLA-DRB5*01:01	55	**0.802**	0.770	0.752	0.748	0.748
HLA-DRB5*01:02	1	**0.987**	0.728	0.762		0.847
Ave per ligand	1325	**0.751**	0.729	0.696		0.725
Ave per allele	42	**0.781**	0.759	0.717		0.762
Tepitope	20	0.747	0.744	0.696	**0.757**	0.755
!Tepitope		**0.811**	0.772	0.736		0.769
