# Supplementary material for: TEPITOPEpan: Extending TEPITOPE for Peptide Binding Prediction Covering over 700 HLA-DR Molecules
Source: PLoS One. 2012 Feb 23;7(2):e30483. doi: 10.1371/journal.pone.0030483 (PMC3285624; doi:10.1371/journal.pone.0030483)
Supplement: Table S4 — Evaluation on identifying binding core. The table shows complexes with known binding cores retrieved from PDB. The first two columns in the table give PDB ID, HLA-DR restriction, bound peptide and experimentally determined binding core, respectively. Twenty distinct structures in terms of allele and peptide sequence are labeled with an asterisk. The last columns give predicted cores of different methods. Predictions of different methods were obtained from their stand-alone packages or web servers. Prediction results based on 20 distinct structures are shown in brackets with an asterisk. Additionally, TEPITOPE can not make prediction for DRB3*01:01 and DRB3*02:01. (PDF) [file pone.0030483.s006.pdf]

Table S4: Evaluation on identifying binding core. The table shows complexes with known binding cores retrieved from PDB. The first two columns in the table give PDB ID, HLA-DR restriction, bound peptide and experimentally determined binding core, respectively. Twenty distinct structures in terms of allele and peptide sequence are labeled with an asterisk. The last columns give predicted cores of different methods. Predictions of different methods were obtained from their stand-alone packages or web servers. Prediction results based on 20 distinct structures are shown in brackets with an asterisk. Additionally, TEPITOPE can not make prediction for DRB3*01:01 and DRB3*02:01.

PDBID	Allele	Peptide	Core	NetMHCIIpan-2.0	NetMHCIIpan-1.0	MultiRTA	TEPITOPE	TEPITOPEpan
1AQD*	DRB1*01:01	VGSDWRFLRGYHQYA	WRFLRGYHQ	WRFLRGYHQ	WRFLRGYHQ	WRFLRGYHQ	WRFLRGYHQ	WRFLRGYHQ
1PYW*	DRB1*01:01	XFVKQNAAALX	FVKQNAAAL	FVKQNAAAL	**VKQNAAALX**	FVKQNAAAL	FVKQNAAAL	FVKQNAAAL
1KLG*	DRB1*01:01	GELIGILNAAKVPAD	IGILNAAKV	**LIGILNAAK**	IGILNAAKV	IGILNAAKV	IGILNAAKV	IGILNAAKV
1KLU*	DRB1*01:01	GELIGTLNAAKVPAD	IGTLNAAKV	IGTLNAAKV	IGTLNAAKV	IGTLNAAKV	IGTLNAAKV	IGTLNAAKV
2FSE*	DRB1*01:01	AGFKGEQGPKGEPG	FKGEQGPKG	FKGEQGPKG	FKGEQGPKG	FKGEQGPKG	FKGEQGPKG	FKGEQGPKG
1SJH*	DRB1*01:01	PEVIPMFSALSEG	VIPMFSALS	VIPMFSALS	VIPMFSALS	VIPMFSALS	VIPMFSALS	VIPMFSALS
1SJE*	DRB1*01:01	PEVIPMFSALSEGATP	VIPMFSALS	VIPMFSALS	VIPMFSALS	VIPMFSALS	VIPMFSALS	VIPMFSALS
1T5W	DRB1*01:01	AAYSDQATPLLLSPR	YSDQATPLL	YSDQATPLL	YSDQATPLL	**SDQATPLLL**	YSDQATPLL	YSDQATPLL
1T5X*	DRB1*01:01	AAYSDQATPLLLSPR	YSDQATPLL	YSDQATPLL	YSDQATPLL	**SDQATPLLL**	YSDQATPLL	YSDQATPLL
2IAN	DRB1*01:01	GELIGTLNAAKVPAD	IGTLNAAKV	IGTLNAAKV	IGTLNAAKV	IGTLNAAKV	IGTLNAAKV	IGTLNAAKV
2IAM	DRB1*01:01	GELIGILNAAKVPAD	IGILNAAKV	**LIGILNAAK**	IGILNAAKV	IGILNAAKV	IGILNAAKV	IGILNAAKV
2IPK*	DRB1*01:01	XPKWVKQNTLKLAT	WVKQNTLKL	WVKQNTLKL	WVKQNTLKL	WVKQNTLKL	WVKQNTLKL	WVKQNTLKL
1FYT*	DRB1*01:01	PKYVKQNTLKLAT	YVKQNTLKL	YVKQNTLKL	YVKQNTLKL	YVKQNTLKL	YVKQNTLKL	YVKQNTLKL
1R5I	DRB1*01:01	PKYVKQNTLKLAT	YVKQNTLKL	YVKQNTLKL	YVKQNTLKL	YVKQNTLKL	YVKQNTLKL	YVKQNTLKL
1HXY	DRB1*01:01	PKYVKQNTLKLAT	YVKQNTLKL	YVKQNTLKL	YVKQNTLKL	YVKQNTLKL	YVKQNTLKL	YVKQNTLKL
1JWM	DRB1*01:01	PKYVKQNTLKLAT	YVKQNTLKL	YVKQNTLKL	YVKQNTLKL	YVKQNTLKL	YVKQNTLKL	YVKQNTLKL
1JWS	DRB1*01:01	PKYVKQNTLKLAT	YVKQNTLKL	YVKQNTLKL	YVKQNTLKL	YVKQNTLKL	YVKQNTLKL	YVKQNTLKL
1JWU	DRB1*01:01	PKYVKQNTLKLAT	YVKQNTLKL	YVKQNTLKL	YVKQNTLKL	YVKQNTLKL	YVKQNTLKL	YVKQNTLKL
1LO5	DRB1*01:01	PKYVKQNTLKLAT	YVKQNTLKL	YVKQNTLKL	YVKQNTLKL	YVKQNTLKL	YVKQNTLKL	YVKQNTLKL
2ICW	DRB1*01:01	PKYVKQNTLKLAT	YVKQNTLKL	YVKQNTLKL	YVKQNTLKL	YVKQNTLKL	YVKQNTLKL	YVKQNTLKL
2OJE	DRB1*01:01	PKYVKQNTLKLAT	YVKQNTLKL	YVKQNTLKL	YVKQNTLKL	YVKQNTLKL	YVKQNTLKL	YVKQNTLKL
2G9H	DRB1*01:01	PKYVKQNTLKLAT	YVKQNTLKL	YVKQNTLKL	YVKQNTLKL	YVKQNTLKL	YVKQNTLKL	YVKQNTLKL
1A6A*	DRB1*03:01	PVSKMRMATPLLMQA	MRMATPLLM	MRMATPLLM	MRMATPLLM	MRMATPLLM	MRMATPLLM	MRMATPLLM
1J8H*	DRB1*04:01	PKYVKQNTLKLAT	YVKQNTLKL	YVKQNTLKL	YVKQNTLKL	YVKQNTLKL	YVKQNTLKL	YVKQNTLKL
2SEB*	DRB1*04:01	AYMRADAAAGGA	MRADAAAGG	**YMRADAAAG**	**YMRADAAAG**	MRADAAAGG	MRADAAAGG	**YMRADAAAG**
1BX2*	DRB1*15:01	ENPVVHFFKNIVTPR	VHFFKNIVT	**VVHFFKNIV**	VHFFKNIVT	VHFFKNIVT	VHFFKNIVT	VHFFKNIVT
1YMM*	DRB1*15:01	ENPVVHFFKNIVTPRGGSGGGGG	VHFFKNIVT	VHFFKNIVT	VHFFKNIVT	VHFFKNIVT	VHFFKNIVT	VHFFKNIVT
2Q6W*	DRB3*01:01	AWRSDEALPLGS	WRSDEALPL	WRSDEALPL	WRSDEALPL	WRSDEALPL		WRSDEALPL
3C5J*	DRB3*02:01	QVIILNHPGQISA	IILNHPGQI	IILNHPGQI	IILNHPGQI	IILNHPGQI		**VIILNHPGQ**
1FV1*	DRB5*01:01	NPVVHFFKNIVTPRTPPPSQ	FKNIVTPRT	**FFKNIVTPR**	FKNIVTPRT	**VHFFKNIVT**	FKNIVTPRT	FKNIVTPRT
1H15*	DRB5*01:01	GGVYHFVKKHVHES	YHFVKKHVH	YHFVKKHVH	**FVKKHVHES**	YHFVKKHVH	YHFVKKHVH	YHFVKKHVH
1ZGL*	DRB5*01:01	VHFFKNIVTPRTPGG	FKNIVTPRT	**FFKNIVTPR**	FKNIVTPRT	**VHFFKNIVT**	FKNIVTPRT	FKNIVTPRT
Results				6 (5*) errors	3 (3*) errors	4 (3*) errors	0 (2 missing)	2 (2*) errors
